# Red Blood Cell Distribution Width, Disease Severity, and Mortality in Hospitalized Patients with SARS-CoV-2 Infection: A Systematic Review and Meta-Analysis

**DOI:** 10.3390/jcm10020286

**Published:** 2021-01-14

**Authors:** Angelo Zinellu, Arduino A. Mangoni

**Affiliations:** 1 Department of Biomedical Sciences, University of Sassari, 07100 Sassari, Italy; azinellu@uniss.it; 2 Discipline of Clinical Pharmacology, College of Medicine and Public Health, Flinders University and Flinders Medical Centre, Adelaide, SA 5042, Australia

**Keywords:** red blood cell distribution width, COVID-19, disease severity, mortality

## Abstract

The identification of biomarkers predicting disease severity and outcomes is the focus of intense research in patients with severe acute respiratory syndrome coronavirus 2 (SARS-CoV-2 infection). Ideally, such biomarkers should be easily derivable from routine tests. We conducted a systematic review and meta-analysis of the predictive role of the red blood cell distribution width (RDW), a routine hematological test, in patients with SARS-CoV-2 infection. We searched the electronic databases PubMed, Web of Science and Scopus, from January 2020 to November 2020, for studies reporting data on the RDW and coronavirus disease 2019 (COVID-19) severity, defined as severe illness or admission to the intensive care unit (ICU), and mortality. Eleven studies in 4901 COVID-19 patients were selected for the meta-analysis. Pooled results showed that the RDW values were significantly higher in patients with severe disease and non-survivors (standard mean difference, SMD = 0.56, 95% CI 0.31 to 0.81, *p* < 0.001). Heterogeneity between studies was extreme (I^2^ = 80.6%; *p* < 0.001). In sensitivity analysis, the effect size was not modified when each study was in turn removed (effect size range, between 0.47 and 0.63). The Begg’s (*p* = 0.53) and Egger’s tests (*p* = 0.52) showed no evidence of publication bias. No significant correlations were observed between SMD and age, gender, whole blood count, end point, study geographic area, or design. Our meta-analysis showed that higher RDW values are significantly associated with COVID-19 severity and mortality. This routine parameter might assist with early risk stratification in patients with SARS-CoV-2 infection.

## 1. Introduction

Coronavirus disease 2019 (COVID-19) is a condition caused by the severe acute respiratory syndrome coronavirus 2 (SARS-CoV-2), the agent responsible for the ongoing global pandemic. Patients with severe forms of COVID-19 exhibit a systemic pro-inflammatory state with associated oxidant stress, coagulation disorders, and multiorgan compromise. This clinical picture often requires intensive care treatment and, potentially, might lead to death [1,2,3]. Proven therapies, pending the widespread distribution of effective vaccines, are limited to the glucocorticoid agent dexamethasone [4,5], with some evidence regarding the antiviral agent remdesivir [6] and the use of anticoagulants [7]. Therefore, the identification of early markers of disease severity would greatly facilitate the selection of COVID-19 patients requiring more aggressive monitoring and management and the judicious use of healthcare resources. Ideally, such markers should be relatively inexpensive and easy to derive, for example from routine tests conducted in this group on admission and throughout the hospitalization. There is good evidence that severe COVID-19 is associated with significant alterations of routine hematological parameters, particularly an increased neutrophil to lymphocyte ratio, thrombocytopaenia, a prolonged pro-thrombin time, and high D-dimer concentrations [8,9]. A routine hematological parameter that has been relatively less studied, in terms of predictive capacity, in this patient population is the red blood cell distribution width (RDW), defined as the coefficient of variation in the volume of circulating red blood cells expressed as a percentage [10,11]. Alterations in the RDW, particularly its elevation that reflects an increased heterogeneity in the volume of red blood cells (anisocytosis), are associated with physiological events, e.g., pregnancy [12], as well as several disease states, particularly cardiovascular and respiratory disease, critical illness, sepsis, and cancer [10]. Elevations of the RDW can be secondary to a reduced clearance of older red blood cells in the spleen and liver and/or a reduced red blood cell production in the bone marrow. The latter has been proposed to occur in the setting of a concomitant increased leukocyte and/or platelet production in pro-inflammatory states [10]. Given that the RDW has been shown to have a good predictive capacity toward adverse clinical outcomes in several acute and chronic disease states [13,14,15,16,17,18,19,20], we conducted a systematic review and meta-analysis of the available evidence on the association between the RDW and measures of disease severity and survival status specifically in patients with SARS-CoV-2 infection.

## 2. Methods

### 2.1. Search Strategy, Eligibility Criteria, and Study Selection

We conducted a literature search, using the terms “RDW” or “red cell distribution width” and “coronavirus disease 19” or “COVID-19,” in the electronic databases PubMed, Web of Science, and Scopus, from January 2020 to November 2020, to identify peer-reviewed studies reporting RDW values, measures of COVID-19 severity, specifically disease severity or admission to the intensive care unit (ICU), and mortality. The references of the retrieved articles were also searched to identify additional studies. Eligibility criteria for study inclusion were the following: (a) Studies reporting continuous data on RDW values in COVID-19 patients; (b) articles investigating COVID-19 patients with different disease severity or clinical outcomes, particularly mortality; (c) articles on adult patients; (d) articles in English; and (e) full-text article available. Two investigators independently screened abstracts to establish relevance. If relevant, the two investigators independently reviewed the full articles. We used the Newcastle-Ottawa scale to assess the quality of each study. The scale evaluates the following components: cohort selection, cohort comparability on the basis of the design or analysis, how the exposure was determined, and how the outcomes of interest were evaluated. Studies with a score of six or more were considered to be of high quality [21]. No specific review protocol was developed.

### 2.2. Statistical Analysis

Standardized mean differences (SMD) were used to build forest plots of continuous data and to evaluate differences in RDW values between COVID-19 patients with low vs. high disease severity or survivor vs. non-survivor status. A *p*-value < 0.05 was considered statistically significant, and 95% confidence intervals (CIs) were reported. When RDW values were reported as median and interquartile range (IQR), the mean and standard deviation were derived as previously described [22]. The Q-statistic was used to test the between-study heterogeneity in SMD values (the significance level was set at *p* < 0.10). Inconsistency across studies was evaluated through the I^2^ statistic (I^2^ < 25%, no heterogeneity; I^2^ between 25% and 50%, moderate heterogeneity; I^2^ between 50% and 75%, large heterogeneity; and I^2^ > 75%, extreme heterogeneity) [23,24]. A random-effects model was used, in the presence of a high heterogeneity, to calculate the pooled SMD values and the corresponding 95% confidence intervals. The influence of each individual study on the overall effect size estimate was investigated using sensitivity analysis, by sequentially excluding one study at a time [25]. The associations between study size and magnitude of effect were analyzed using the Begg’s adjusted rank correlation test and the Egger’s regression asymmetry test, at the *p* < 0.05 level of significance, to assess the presence of potential publication bias [26,27]. The Duval and Tweedie “trim and fill” procedure was used to further test the possible effect of publication bias [28]. This method recalculates a pooled SMD by incorporating the hypothetical missing studies as though they actually existed, to augment the observed data so that the funnel plot is more symmetric. Statistical analyses were performed using Stata 14 (STATA Corp., College Station, TX, USA). The study was fully compliant with the PRISMA statement regarding the reporting of systematic reviews and meta-analyses [29].

## 3. Results

### 3.1. Literature Search and Study Selection

**Figure 1:**
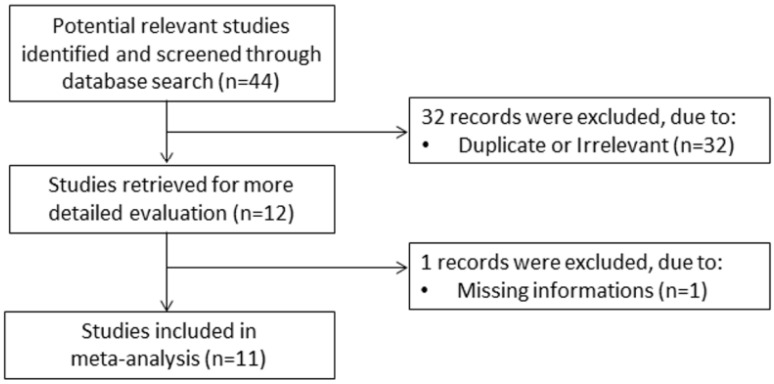
Flow chart of study selection.

A flow chart describing the screening process is presented in Figure 1. We initially identified 44 studies. A total of 32 studies were excluded because they were either duplicates or irrelevant. After a full-text review of the remaining 12 articles, one further study was excluded because of missing information. Thus, 11 studies were included in the meta-analysis [30,31,32,33,34,35,36,37,38,39,40]. Their characteristics are presented in Table 1. A total of 4901 COVID-19 patients were studied, 4247 (50% males, mean age 52 years) with low severity or who survived and 654 (57% males, mean age 66 years) with high severity or who died. Two studies were prospective [34,36], while the remaining nine were retrospective [30,31,32,33,35,37,38,39,40]. Six studies were performed in Asia [30,33,35,38,39,40], three in Europe [32,36,37], and two in America [31,34]. End points included disease severity based on current clinical guidelines in six studies [30,33,34,35,39,40], survival in three [31,36,37], and transfer to ICU in two [32,38].

### 3.2. Meta-Analysis

**Figure 2:**
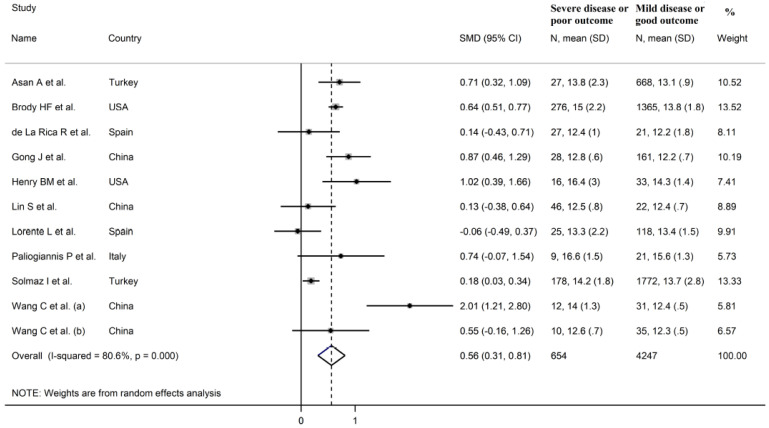
Forest plot of studies examining red blood cell distribution width (RDW) values in patients with COVID-19. The diamond represents the point estimate and confidence intervals after combining and averaging all the individual studies together. The vertical line through the vertical points of the diamond represents the point estimate of the averaged studies.

**Figure 3:**
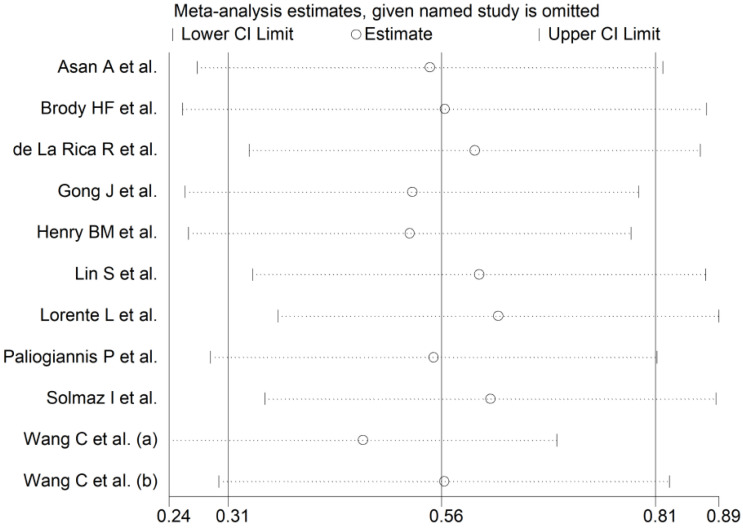
Sensitivity analysis of the association between RDW and COVID-19. The influence of individual studies on the overall standardized mean difference (SMD) is shown. The middle vertical axis indicates the overall SMD and the two vertical axes indicate the 95% confidence intervals (CI). Hollow circles represent the pooled SMD when the remaining study is omitted from the meta-analysis. The two ends of each broken line represent the 95% CIs.

**Figure 4:**
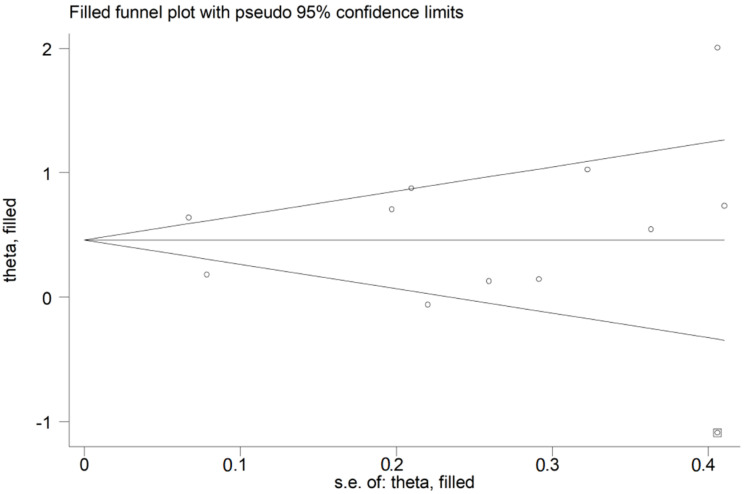
Funnel plot of studies investigating low vs. high severity or survivor vs. non-survivor status after trimming and filling. Dummy studies and genuine studies are represented by enclosed circles and free circles, respectively.

**Figure 5:**
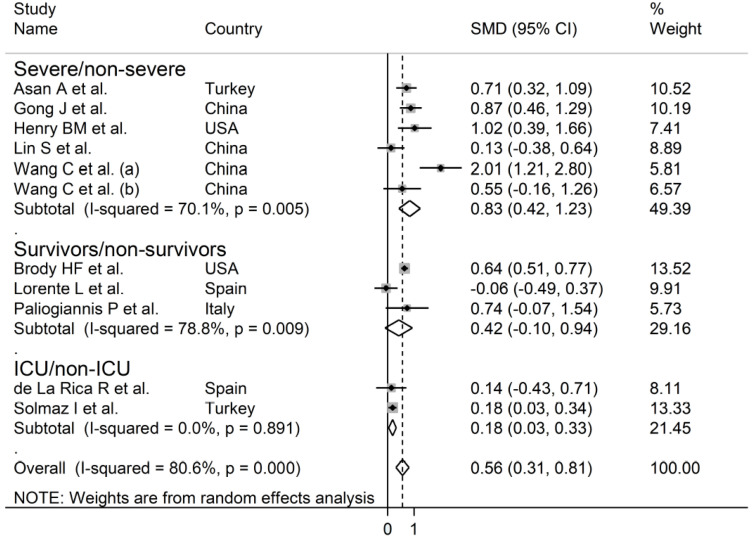
Forest plot of studies examining RDW values in patients with COVID-19 according to disease severity or survival status. The diamond represents the point estimate and confidence intervals after combining and averaging all the individual studies together. The vertical line through the vertical points of the diamond represents the point estimate of the averaged studies.

**Figure 6:**
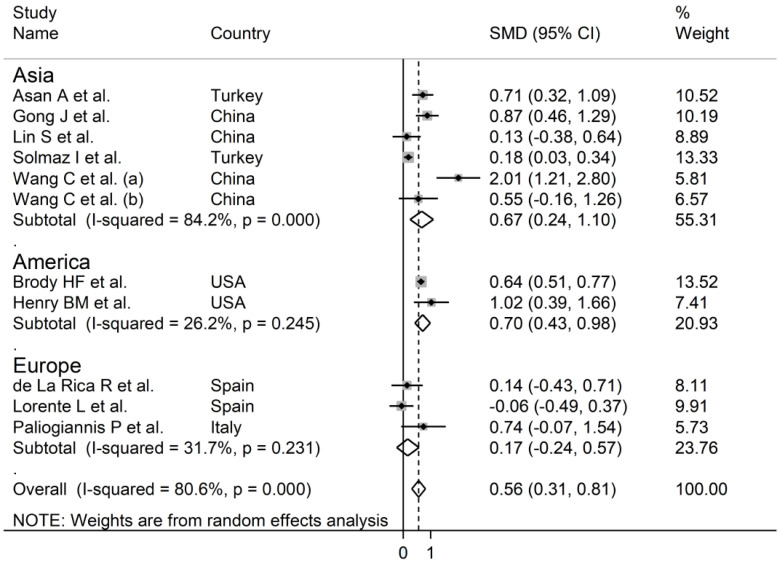
Forest plot of studies examining RDW values in patients with COVID-19 according to the geographic area where the study was conducted. The diamond represents the point estimate and confidence intervals after combining and averaging all the individual studies together. The vertical line through the vertical points of the diamond represents the point estimate of the averaged studies.

**Figure 7:**
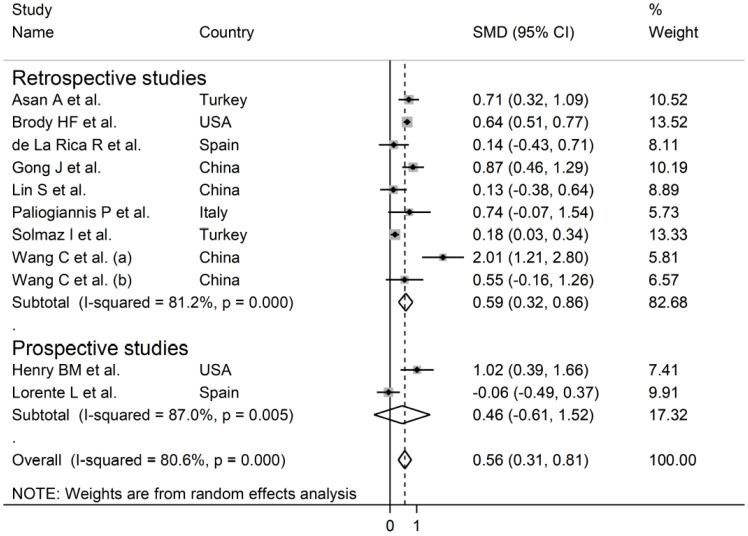
Forest plot of studies examining RDW values in patients with COVID-19 according to the study design (prospective vs. retrospective). The diamond represents the point estimate and confidence intervals after combining and averaging all the individual studies together. The vertical line through the vertical points of the diamond represents the point estimate of the averaged studies.

The overall SMD in RDW values between COVID-19 patients with mild vs. severe disease or survivor vs. non-survivor status in the 11 studies is shown in Figure 2. In 10 of these studies, patients with severe disease or non-survivor status displayed higher RDW values when compared to those with mild disease or survivor status (mean difference range, 0.13 to 2.01) [30,31,32,33,34,35,37,38,39,40]. However, the difference was not statistically significant in four studies [32,35,37,40]. In the remaining study, the RDW values were mildly, non-significantly, higher in patients with mild disease or survivor status (mean difference, −0.06) [36]. The pooled results confirmed that the RDW values were significantly higher in patients with severe disease or non-survivor status (SMD 0.56, 95% CI 0.31 to 0.81, *p* < 0.001) (Figure 2). Extreme heterogeneity between studies was observed (I^2^ = 80.6%; *p* < 0.001). Sensitivity analysis showed that the effect size was not affected when each study was in turn removed (effect size range, between 0.47 and 0.63) (Figure 3). The Begg’s (*p* = 0.53) and Egger’s tests (*p* = 0.52) showed no evidence of publication bias. However, the trim-and-fill method identified one potential missing study to add on the left side of the funnel plot to ensure symmetry (Figure 4). The adjusted SMD value was attenuated but remained statistically significant (SMD 0.47, 95% CI 0.21 to 0.74, *p* = 0.001). To explore the possible contributors to the between-study variance, we investigated the effects of age, gender, publication geographic area, end points, study design (retrospective vs. prospective) and the inflammation biomarker white blood cell (WBC) count on the SMD by univariate meta-regression analysis. No statistically significant correlations were observed between the SMD and age (t = −0.09, *p* = 0.93), gender (t = 0.56, *p* = 0.59) and WBC (t = 0.56, *p* = 0.59). The pooled SMD value for mild/severe disease studies (0.83, 95% CI 0.42 to 1.23, *p* < 0.001; I^2^ = 70.1%, *p* = 0.005) was higher than that observed in survivor/non-survivor status studies (0.42, 95% CI −0.10 to 0.94, *p* = 0.11; I^2^ = 78.8%, *p* = 0.009) and in ICU/non-ICU studies (0.18, 95% CI 0.03 to 0.33, *p* = 0.017; I^2^ = 0.0%, *p* = 0.89) however the difference was not statistically significant by meta regression analysis (t = −1.30, *p* = 0.22, Figure 5). In addition, the pooled SMD value in American studies (0.70, 95% CI 0.43 to 0.98, *p* = 0.002; I^2^ = 26.2%, *p* = 0.25) was lower than that observed in Asian (0.67, 95% CI 0.24 to 1.10, *p* < 0.001; I^2^ = 84.2%, *p* < 0.001) and European studies (0.17, 95% CI −0.24 to 0.57, *p* = 0.44; I^2^ = 31.7%, *p* = 0.23) although, also in this case, the difference was not statistically significant (t = −0.04, *p* = 0.97, Figure 6). However, a relatively lower heterogeneity was observed in ICU/non-ICU, I^2^ = 0.0%, American, I^2^ = 26.2%, and European, I^2^ = 31.7%, studies. Finally, non-significant differences (t = −0.42, *p* = 0.68) were also observed between pooled SMD values from retrospective (0.59, 95% CI 0.32 to 0.86, *p* < 0.001; I^2^ = 81.2%, *p* < 0.001) and prospective studies (0.46, 95% CI −0.61 to 1.52, *p* = 0.40; I^2^ = 80.6%, *p* < 0.001, Figure 7).

## 4. Discussion

In our systematic review and meta-analysis, the RDW values were significantly higher in COVID-19 patients with severe disease, i.e., those with severe clinical manifestations of the disease or requiring admission to the ICU, or non-survivor status when compared to patients with mild disease or survivor status. Despite the extreme between-study heterogeneity sensitivity analysis showed that the overall effect size was not significantly affected when individual studies were removed. Furthermore, there was no evidence of publication bias. Notably, the SMD was not significantly associated with several variables, such as age, gender, WBC, type of end point studied (disease severity, admission to ICU, or survival), study geographic area or design (retrospective vs. prospective). Our meta-analysis provides useful information regarding the available evidence on the association between the RDW and a number of different clinical endpoints, i.e., COVID-19 severity, transfer to ICU, or survival status. This might further assist with early risk stratification and selection of personalized treatment strategies.

The RDW, a parameter reported in the vast majority of standard hematological tests performed in primary care and hospital settings across the globe, reflects the degree of heterogeneity of the volume of red blood cells. The presence of a relatively high degree of heterogeneity is known as anisocytosis [11]. The RDW is typically calculated as the standard deviation of the volume of red blood cells divided by their mean corpuscular volume. The obtained value is then multiplied by 100, which leads to the final RDW value, expressed as a percentage [10,11]. While RDW values below the normal range are extremely rare in clinical practice, and of dubious significance, values above this range are commonly observed in a number of physiological and pathological states [10,12]. Furthermore, and perhaps more importantly, higher RDW values have been shown to independently predict adverse clinical outcomes in systematic reviews and meta-analyses focusing on specific disease states, particularly hematological malignancies, solid cancers, heart failure, ischemic heart disease, stroke, critical illness, and sepsis [13,14,15,16,17,18,19]. However, it is not possible to compare the effect size between our meta-analysis and these studies as the results in the latter were expressed as risk ratio, odds ratio, or hazard ratio. Therefore, while the available evidence suggests that higher RDW values represent a non-specific marker of disease, which likely reflects alterations in red blood cell turnover in response to an excess production of other cells in the bone marrow in the context of systemic inflammation, this parameter might assist in management decisions in hospitalized patients with COVID-19, a relatively new and potentially lethal viral disease with limited therapeutic options. For example, higher RDW values on admission might help identify those patients that are more at risk of developing severe clinical manifestations, potentially requiring intensive monitoring and early transfer to an ICU setting. This might provide a number of benefits in terms of patient outcomes, length of stay, and resource allocation, particularly in situations of high patient load and staff shortages.

The exact mechanisms underlying the increased RDW values in severe COVID-19 patients and in those who succumb to the disease are unclear. However, the presence of a significant, systemic, pro-inflammatory, and pro-oxidant state in this cohort might play a role. For example, the presence of reduced circulating antioxidant factors has been shown to be negatively associated with the RDW, possibly through an excessive turnover of red blood cells [41,42]. Furthermore, the presence of a pro-inflammatory state, and the release of specific cytokines, can inhibit the synthesis or the activity of erythropoietin, the hormone primarily responsible for the production of red blood cells in the bone marrow, alter iron metabolism and reduce survival of mature red blood cells, all factors contributing to an elevation of the RDW [43,44]. The co-existence of pro-inflammatory and pro-oxidant disease states that are associated with both increased RDW values and poor COVID-19 outcomes, e.g., obesity, chronic kidney disease and diabetes [45,46,47], might explain, at least in part, the association between the RDW and COVID-19 disease severity and mortality.

A systematic review and meta-analysis has previously investigated whether RDW values predict severe disease in patients with SARS-CoV-2 infection [48]. While the reported weighted mean difference between patients with and without severe disease are comparable with those observed in our study (0.69 vs. 0.56%) only three studies were assessed, including a study published in MedRxiv but not peer reviewed. As this study remains unpublished following peer review, at the time our literature search was conducted, it was not included in our systematic review and meta-analysis [49]. Furthermore, given the substantially larger number of studies identified (*n* = 11), in our meta-analysis we also assessed the SMD values according to specific end-points (disease severity, ICU admission, and death), geographical area were the studies were conducted, and study design (prospective vs. retrospective). While the extreme between-study heterogeneity represents a potential limitation, which curtails the interpretation of the results, the overall effect size was not significantly affected in sensitivity analysis and, reassuringly, no evidence of publication bias was observed. Despite conducting further analyses to identify the potential source of heterogeneity, no significant associations were observed between the SMD values and a number of clinical, demographic, and study characteristics. However, sub-group analysis showed relatively lower values of heterogeneity in ICU/non-ICU, American, and European studies, suggesting that specific end points and publication geographic area may, at least partially, contribute to the observed between-study variance. It is possible that other, unreported, factors might have contributed to the observed heterogeneity. One such factor is the method used for red blood cell analysis and RDW measurement. An excellent review on the topic highlights this as a potential source of variability between different studies that use specific analyzers and advocates an increasing adherence by the manufacturers to the recommendations issued by the International Council for Standardization in Hematology regarding the standardization of red blood cell distribution curve analysis [10,50].

In conclusion, our systematic review and meta-analysis has shown that higher RDW values are significantly associated with high disease severity and mortality in patients with SARS-CoV-2 infection. The RDW, singly or used in combination with clinical and demographic parameters and/or other markers of inflammation or altered hematological hemostasis, might represent a relatively inexpensive and easy to derive biomarker to guide early management decisions in patients with COVID-19.

## Figures and Tables

**Table 1:** Characteristics of the selected studies in COVID-19 patients, according to disease severity and survival status.

				Mild Disease or Survivor				Severe Disease or Non-Survivor			
First Author, Country	Study Design	Outcome	NOS (Stars)	n	Age (Years)	Gender (M/F)	RDW (%, Mean ± SD)	n	Age (Years)	Gender (M/F)	RDW (%, Mean ± SD)
Asan A., et al. [30], Turkey	R	Severe Non-severe	7	668	41	316/352	13.1 ± 0.9	27	69	15/12	13.8 ± 2.3
Brody H.F., et al. [31], USA	R	Survivor Non-survivor	8	1365	60	723/642	13.8 ± 1.8	276	75	163/113	15.0 ± 2.2
de La Rica R., et al. [32], Spain	R	ICU Non-ICU	7	21	66	18/3	12.2 ± 1.8	27	66	14/13	12.4 ± 1.0
Gong J., et al. [33], China	R	Severe Non-severe	7	161	45	72/89	12.2 ± 0.7	28	64	16/12	12.8 ± 0.6
Henry B.M., et al. [34], USA	P	Severe Non-severe	7	33	49	19/14	14.3 ± 1.4	16	63	10/6	16.4 ± 3.0
Lin S., et al. [35], China	R	Severe Non-severe	7	22	44	11/11	12.4 ± 0.7	46	56	29/17	12.5 ± 0.8
Lorente L., et al. [36], Spain	P	Survivor Non-survivor	7	118	64	53/65	13.4 ± 1.5	25	71	7/18	13.3 ± 2.2
Paliogiannis P., et al. [37], Italy	R	Survivor Non-survivor	7	21	64	12/9	15.6 ± 1.3	9	82	8/1	16.6 ± 1.5
Solmaz I., et al. [38], Turkey	R	ICU Non-ICU	7	1772	47	881/891	13.7 ± 2.8	178	66	96/82	14.2 ± 1.8
Wang C., et al. (a) [39], China	R	Severe Non-severe	7	31	56	18/13	12.4 ± 0.5	12	67	7/5	14.0 ± 1.3
Wang C., et al. (b) [40], China	R	Severe Non-severe	7	35	38	17/18	12.3 ± 0.5	10	43	6/4	12.6 ± 0.7

ICU: intensive care unit; Non-severe: patients with mild or moderate disease; NOS: Newcastle-Ottawa quality assessment scale for case-control studies; P: prospective; R: retrospective; Severe: patients with severe or critical disease. RDW: red blood cell distribution width.

## References

[1] Romagnoli S., Peris A., De Gaudio A.R., Geppetti P. (2020). SARS-CoV-2 and COVID-19: From the Bench to the Bedside. Physiol. Rev..

[2] Dhama K., Khan S., Tiwari R., Sircar S., Bhat S., Malik Y.S., Singh K.P., Chaicumpa W., Bonilla-Aldana D.K., Rodriguez-Morales A.J. (2020). Coronavirus Disease 2019-COVID-19. Clin. Microbiol. Rev..

[3] Leisman D.E., Ronner L., Pinotti R., Taylor M.D., Sinha P., Calfee C.S., Hirayama A.V., Mastroiani F., Turtle C.J., Harhay M.O. (2020). Cytokine elevation in severe and critical COVID-19: A rapid systematic review, meta-analysis, and comparison with other inflammatory syndromes. Lancet Respir. Med..

[4] Group R.C., Horby P., Lim W.S., Emberson J.R., Mafham M., Bell J.L., Linsell L., Staplin N., Brightling C., Ustianowski A. (2020). Dexamethasone in Hospitalized Patients with Covid-19—Preliminary Report. N. Engl. J. Med..

[5] Siemieniuk R.A., Bartoszko J.J., Ge L., Zeraatkar D., Izcovich A., Kum E., Pardo-Hernandez H., Rochwerg B., Lamontagne F., Han M.A. (2020). Drug treatments for covid-19: Living systematic review and network meta-analysis. BMJ.

[6] Beigel J.H., Tomashek K.M., Dodd L.E., Mehta A.K., Zingman B.S., Kalil A.C., Hohmann E., Chu H.Y., Luetkemeyer A., Kline S. (2020). Remdesivir for the Treatment of Covid-19—Final Report. N. Engl. J. Med..

[7] Kamel A.M., Sobhy M., Magdy N., Sabry N., Farid S. (2020). Anticoagulation outcomes in hospitalized Covid-19 patients: A systematic review and meta-analysis of case-control and cohort studies. Rev. Med. Virol..

[8] Liao D., Zhou F., Luo L., Xu M., Wang H., Xia J., Gao Y., Cai L., Wang Z., Yin P. (2020). Haematological characteristics and risk factors in the classification and prognosis evaluation of COVID-19: A retrospective cohort study. Lancet Haematol..

[9] Paliogiannis P., Mangoni A.A., Dettori P., Nasrallah G.K., Pintus G., Zinellu A. (2020). D-Dimer Concentrations and COVID-19 Severity: A Systematic Review and Meta-Analysis. Front. Public Health.

[10] Salvagno G.L., Sanchis-Gomar F., Picanza A., Lippi G. (2015). Red blood cell distribution width: A simple parameter with multiple clinical applications. Crit. Rev. Clin. Lab. Sci..

[11] Ford J. (2013). Red blood cell morphology. Int. J. Lab. Hematol..

[12] Paliogiannis P., Zinellu A., Mangoni A.A., Capobianco G., Dessole S., Cherchi P.L., Carru C. (2018). Red blood cell distribution width in pregnancy: A systematic review. Biochem. Med. (Zagreb).

[13] Ai L., Mu S., Hu Y. (2018). Prognostic role of RDW in hematological malignancies: A systematic review and meta-analysis. Cancer Cell Int..

[14] Hu L., Li M., Ding Y., Pu L., Liu J., Xie J., Cabanero M., Li J., Xiang R., Xiong S. (2017). Prognostic value of RDW in cancers: A systematic review and meta-analysis. Oncotarget.

[15] Huang Y.L., Hu Z.D., Liu S.J., Sun Y., Qin Q., Qin B.D., Zhang W.W., Zhang J.R., Zhong R.Q., Deng A.M. (2014). Prognostic value of red blood cell distribution width for patients with heart failure: A systematic review and meta-analysis of cohort studies. PLoS ONE.

[16] Su C., Liao L.Z., Song Y., Xu Z.W., Mei W.Y. (2014). The role of red blood cell distribution width in mortality and cardiovascular risk among patients with coronary artery diseases: A systematic review and meta-analysis. J. Thorac. Dis..

[17] Song S.Y., Hua C., Dornbors D., Kang R.J., Zhao X.X., Du X., He W., Ding Y.C., Meng R. (2019). Baseline Red Blood Cell Distribution Width as a Predictor of Stroke Occurrence and Outcome: A Comprehensive Meta-Analysis of 31 Studies. Front. Neurol..

[18] Luo R., Hu J., Jiang L., Zhang M. (2016). Prognostic Value of Red Blood Cell Distribution Width in Non-Cardiovascular Critically or Acutely Patients: A Systematic Review. PLoS ONE.

[19] Zhang L., Yu C.H., Guo K.P., Huang C.Z., Mo L.Y. (2020). Prognostic role of red blood cell distribution width in patients with sepsis: A systematic review and meta-analysis. BMC Immunol..

[20] Triantafyllidi H., Palaiodimos L., Ikonomidis I., Schoinas A., Pavlidis G., Trivilou P., Lekakis J. (2016). The independent association of two “priceless” parameters: Pulse pressure and red cell distribution width in recently diagnosed hypertensive patients. Hell. J. Cardiol..

[21] Wells G.A., Shea B., O’Connell D., Peterson J., Welch V., Losos M., Tugwell P. The Newcastle-Ottawa Scale (NOS) for Assessing the Quality of Nonrandomised Studies in Meta-Analyses. http://www.ohri.ca/programs/clinical_epidemiology/oxford.asp.

[22] Wan X., Wang W., Liu J., Tong T. (2014). Estimating the sample mean and standard deviation from the sample size, median, range and/or interquartile range. BMC Med. Res. Methodol..

[23] Bowden J., Tierney J.F., Copas A.J., Burdett S. (2011). Quantifying, displaying and accounting for heterogeneity in the meta-analysis of RCTs using standard and generalised Q statistics. BMC Med. Res. Methodol..

[24] Higgins J.P., Thompson S.G. (2002). Quantifying heterogeneity in a meta-analysis. Stat. Med..

[25] Tobias A. (1999). Assessing the influence of a single study in the meta-analysis estimate. Stata Tech. Bull..

[26] Begg C.B., Mazumdar M. (1994). Operating characteristics of a rank correlation test for publication bias. Biometrics.

[27] Sterne J.A., Egger M. (2001). Funnel plots for detecting bias in meta-analysis: Guidelines on choice of axis. J. Clin. Epidemiol..

[28] Duval S., Tweedie R. (2000). Trim and fill: A simple funnel-plot-based method of testing and adjusting for publication bias in meta-analysis. Biometrics.

[29] Liberati A., Altman D.G., Tetzlaff J., Mulrow C., Gotzsche P.C., Ioannidis J.P., Clarke M., Devereaux P.J., Kleijnen J., Moher D. (2009). The PRISMA statement for reporting systematic reviews and meta-analyses of studies that evaluate healthcare interventions: Explanation and elaboration. BMJ.

[30] Asan A., UstUnda G.Y., Koca N., ŞİmŞek A., Sayan H.E., Parildar H., Dalyan C.B., Huysal K. (2020). Do initial hematologic indices predict the severity of COVID-19 patients?. Turk. J. Med. Sci..

[31] Foy B.H., Carlson J.C.T., Reinertsen E., Padros I.V.R., Lopez R.P., Palanques-Tost E., Mow C., Westover M.B., Aguirre A.D., Higgins J.M. (2020). Association of Red Blood Cell Distribution Width With Mortality Risk in Hospitalized Adults with SARS-CoV-2 Infection. JAMA Netw. Open.

[32] de la Rica R., Borges M., Aranda M., Del Castillo A., Socias A., Payeras A., Rialp G., Socias L., Masmiquel L., Gonzalez-Freire M. (2020). Low Albumin Levels Are Associated with Poorer Outcomes in a Case Series of COVID-19 Patients in Spain: A Retrospective Cohort Study. Microorganisms.

[33] Gong J., Ou J., Qiu X., Jie Y., Chen Y., Yuan L., Cao J., Tan M., Xu W., Zheng F. (2020). A Tool for Early Prediction of Severe Coronavirus Disease 2019 (COVID-19): A Multicenter Study Using the Risk Nomogram in Wuhan and Guangdong, China. Clin. Infect. Dis..

[34] Henry B.M., Benoit J.L., Benoit S., Pulvino C., Berger B.A., Olivera M.H.S., Crutchfield C.A., Lippi G. (2020). Red Blood Cell Distribution Width (RDW) Predicts COVID-19 Severity: A Prospective, Observational Study from the Cincinnati SARS-CoV-2 Emergency Department Cohort. Diagnostics (Basel).

[35] Lin S., Mao W., Zou Q., Lu S., Zheng S. (2020). Associations between hematological parameters and disease severity in patients with SARS-CoV-2 infection. J. Clin. Lab. Anal..

[36] Lorente L., Martin M.M., Argueso M., Sole-Violan J., Perez A., Ramos J., Ramos-Gomez L., Lopez S., Franco A., Gonzalez-Rivero A.F. (2020). Association between red blood cell distribution width and mortality of COVID-19 patients. Anaesth. Crit. Care Pain Med..

[37] Paliogiannis P., Zinellu A., Scano V., Mulas G., De Riu G., Pascale R.M., Arru L.B., Carru C., Pirina P., Mangoni A.A. (2020). Laboratory test alterations in patients with COVID-19 and non COVID-19 interstitial pneumonia: A preliminary report. J. Infect. Dev. Ctries..

[38] Solmaz I., Ozcaylak S., Alakus O.F., Kilic J., Kalin B.S., Guven M., Arac S., Akkoc H. (2020). Risk factors affecting ICU admission in COVID-19 patients; Could air temperature be an effective factor?. Int. J. Clin. Pr..

[39] Wang C., Zhang H., Cao X., Deng R., Ye Y., Fu Z., Gou L., Shao F., Li J., Fu W. (2020). Red cell distribution width (RDW): A prognostic indicator of severe COVID-19. Ann. Transl. Med..

[40] Wang C., Deng R., Gou L., Fu Z., Zhang X., Shao F., Wang G., Fu W., Xiao J., Ding X. (2020). Preliminary study to identify severe from moderate cases of COVID-19 using combined hematology parameters. Ann. Transl. Med..

[41] Friedman J.S., Lopez M.F., Fleming M.D., Rivera A., Martin F.M., Welsh M.L., Boyd A., Doctrow S.R., Burakoff S.J. (2004). SOD2-deficiency anemia: Protein oxidation and altered protein expression reveal targets of damage, stress response, and antioxidant responsiveness. Blood.

[42] Semba R.D., Patel K.V., Ferrucci L., Sun K., Roy C.N., Guralnik J.M., Fried L.P. (2010). Serum antioxidants and inflammation predict red cell distribution width in older women: The Women’s Health and Aging Study I. Clin. Nutr..

[43] Weiss G., Goodnough L.T. (2005). Anemia of chronic disease. N. Engl. J. Med..

[44] Kiefer C.R., Snyder L.M. (2000). Oxidation and erythrocyte senescence. Curr. Opin. Hematol..

[45] Palaiodimos L., Kokkinidis D.G., Li W., Karamanis D., Ognibene J., Arora S., Southern W.N., Mantzoros C.S. (2020). Severe obesity, increasing age and male sex are independently associated with worse in-hospital outcomes, and higher in-hospital mortality, in a cohort of patients with COVID-19 in the Bronx, New York. Metabolism.

[46] Petrilli C.M., Jones S.A., Yang J., Rajagopalan H., O’Donnell L., Chernyak Y., Tobin K.A., Cerfolio R.J., Francois F., Horwitz L.I. (2020). Factors associated with hospital admission and critical illness among 5279 people with coronavirus disease 2019 in New York City: Prospective cohort study. BMJ.

[47] Palaiodimos L., Chamorro-Pareja N., Karamanis D., Li W., Zavras P.D., Chang K.M., Mathias P., Kokkinidis D.G. (2020). Diabetes is associated with increased risk for in-hospital mortality in patients with COVID-19: A systematic review and meta-analysis comprising 18,506 patients. Hormones (Athens).

[48] Lippi G., Henry B.M., Sanchis-Gomar F. (2020). Red Blood Cell Distribution Is a Significant Predictor of Severe Illness in Coronavirus Disease 2019. Acta Haematol..

[49] Levy T.J., Richardson S., Coppa K., Barnaby D.P., McGinn T., Becker L.B., Davidson K.W., Cohen S.L., Hirsch J.S., Zanos T. (2020). Development and Validation of a Survival Calculator for Hospitalized Patients with COVID-19. medRxiv.

[50] England J.M., Rowan R.M., Bull B.S., Coulter W.H., Groner W., Jones A.R., Koepke J.A., Lewis S.M., Shinton N.K., Thom R. (1990). ICSH recommendations for the analysis of red cell, white cell and platelet size distribution curves. Methods for fitting a single reference distribution and assessing its goodness of fit. International Committee for Standardization in Haematology. ICSH Expert Panel on Cytometry. Clin. Lab. Haematol..

